# The CTR hydrophobic residues of Nem1 catalytic subunit are required to form a protein phosphatase complex with Spo7 to activate yeast Pah1 PA phosphatase

**DOI:** 10.1016/j.jbc.2024.108003

**Published:** 2024-11-17

**Authors:** Ruta Jog, Gil-Soo Han, George M. Carman

**Affiliations:** Department of Food Science and the Rutgers Center for Lipid Research, Rutgers University, New Brunswick, New Jersey, USA

## Abstract

The Nem1–Spo7 phosphatase complex plays a key role in lipid metabolism as an activator of Pah1 phosphatidate phosphatase, which produces diacylglycerol for the synthesis of triacylglycerol and membrane phospholipids. For dephosphorylation of Pah1, the Nem1 catalytic subunit requires Spo7 for the recruitment of the protein substrate and interacts with the regulatory subunit through its conserved region (residues 251–446). In this work, we found that the Nem1 C-terminal region (CTR) (residues 414–436), which flanks the haloacid dehalogenase–like catalytic domain (residues 251–413), contains the conserved hydrophobic residues (L414, L415, L417, L418, L421, V430, L434, and L436) that are necessary for the complex formation with Spo7. AlphaFold predicts that some CTR residues of Nem1 interact with Spo7 conserved regions, whereas some residues interact with the haloacid dehalogenase–like domain. By site-directed mutagenesis, Nem1 variants were constructed to lack (Δ(414–446)) or substitute alanines (8A) and arginines (8R) for the hydrophobic residues. When co-expressed with Spo7, the CTR variants of Nem1 did not form a complex with Spo7. In addition, the Nem1 variants were incapable of catalyzing the dephosphorylation of Pah1 in the presence of Spo7. Moreover, the Nem1 variants expressed in *nem1*Δ cells did not complement the phenotypes characteristic of a defect in the Nem1-Spo7/Pah1 phosphatase cascade function (*e.g.*, lipid synthesis, lipid droplet formation, and phospholipid biosynthetic gene expression). These findings support that Nem1 interacts with Spo7 through its CTR hydrophobic residues to form a phosphatase complex for catalytic activity and physiological functions.

Nem1 (nuclear envelope morphology) in the yeast *Saccharomyces cerevisiae* was originally identified by its genetic interaction with the nucleoporin Nup84 and shown to play an essential role in the formation of spherical nuclear morphology (1). Accordingly, cells lacking Nem1 exhibit an aberrant expansion of nuclear/endoplasmic reticulum (ER) membrane (1). The *nem1*Δ cells also display defects in diverse cellular processes such as triacylglycerol (TAG) synthesis (2), lipid droplet formation (3, 4, 5), sporulation (1), autophagy (5, 6, 7, 8), and mitophagy (9). The physiological defects of the *nem1*Δ cells stem from the lack of the Nem1 protein phosphatase activity to dephosphorylate and thereby activate Pah1 phosphatidate (PA) phosphatase (10, 11), which catalyzes the dephosphorylation of PA to produce diacylglycerol (DAG) at the nuclear/ER membrane (12) (Fig. 1).

**Figure 1:**
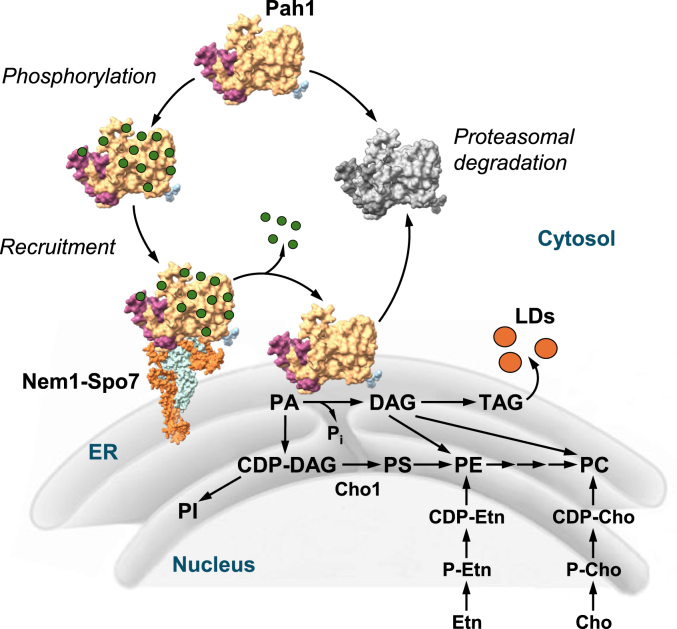
**Model for the Nem1-Spo7**–**mediated activation of Pah1 for its role in lipid synthesis at the nuclear/ER membrane.** Following its expression, Pah1 in the cytosol is unstable and highly phosphorylated for protection against proteasomal degradation. Phosphorylated Pah1 is stable but functionally inactive due to its cytosolic localization. The Nem1–Spo7 complex recruits and dephosphorylates Pah1 at the nuclear/ER membrane. Following its dephosphorylation, Pah1 associates with the membrane and catalyzes the dephosphorylation of PA to produce DAG, which is then acylated to TAG that is stored in lipid droplets. Under the conditions of choline and/or ethanolamine supplementation, the DAG is also used for the synthesis of phosphatidylcholine and/or phosphatidylethanolamine *via* CDP-choline and/or CDP-ethanolamine, respectively. Following rounds of catalysis, Pah1 dissociating from the nuclear/ER membrane is subject to proteasomal degradation (indicated by *gray shading*). AlphaFold (13) structures of Pah1 (N-LIP, *purple*; HAD-like, *gold*) and the Nem1 (*orange*)-Spo7 (*light blue*) complex are depicted. For simplicity, some domains/regions of the proteins are not shown. *Green* dot represent a phosphate group. Refs. (14, 15) illustrate comprehensive pathways for lipid synthesis in *S. cerevisiae*. LDs, lipid droplets; PI, phosphatidylinositol; PS, phosphatidylserine; PE, phosphatidylethanolamine; PC, phosphatidylcholine; Etn, ethanolamine; Cho, choline; HAD, haloacid dehalogenase; DAG, diacylglycerol; TAG, triacylglycerol.

The substrate and product of PA phosphatase are key intermediates in lipid synthesis (Fig. 1). The substrate PA is the precursor of major membrane phospholipids synthesized *via* CDP-DAG, whereas the product DAG is a direct precursor of TAG that is stored in lipid droplets (14, 15, 16). When cells are supplemented with the phospholipid precursors choline and ethanolamine, the DAG produced by PA phosphatase is also used for the synthesis of phosphatidylcholine and phosphatidylethanolamine *via* CDP-choline and CDP-ethanolamine, respectively (14, 15, 16). In addition to their uses for lipid synthesis, PA and DAG play roles as signaling molecules. For example, PA is a key controller in the transcription of the UAS_INO_-containing phospholipid synthesis genes *via* the Henry (Opi1/Ino2-Ino4) regulatory circuit (14, 15, 16, 17), regulates target of rapamycin complex 1 signaling (18, 19, 20), and serves as a pH sensor that links membrane biogenesis to nutrient availability and cell metabolism (21). DAG, along with phosphatidylserine, activates Pkc1 protein kinase C (22) to phosphorylate and regulate lipid metabolic enzymes (14) that include Nem1 (23) and Pah1 (24). Moreover, PA and DAG affect membrane-associated processes through their physical effects on the structure of phospholipid bilayers (25, 26, 27, 28, 29, 30, 31). Thus, the dephosphorylation-mediated activation of Pah1 by Nem1 is a key step in the regulation of the PA-DAG balance, which has multiple regulatory effects on lipid synthesis and cell physiology (16). As can be expected from the requirement of Nem1 for Pah1 function, both *nem1*Δ and *pah1*Δ cells display the same phenotypes (16), which include elevated levels of PA and phospholipids, the aberrant expansion of the nuclear/ER membrane, and reduced levels of TAG and lipid droplets (2, 10, 12, 32, 33, 34).

Spo7, which was originally discovered as a component required for sporulation (35), was identified as a protein forming an obligate complex with Nem1 to maintain the nuclear morphology (1). The complex formation, which is mediated by three conserved regions (CR1, CR2, and CR3) of Spo7 (36, 37), is required to stabilize Nem1 and facilitate its catalytic function to dephosphorylate Pah1 (10, 11, 38, 39, 40). The nuclear/ER membrane-associated Spo7, as the regulatory subunit of Nem1-Spo7, recruits cytosolic Pah1, which is highly phosphorylated (41), for its dephosphorylation by Nem1 (10, 38) (Fig. 1). For the recruitment, the basic tail of Spo7 (42) interacts with the acidic tail of Pah1 (40). The requirement of Spo7 for Nem1 activity on Pah1 is emphasized by the *spo7*Δ phenotypes that resemble those imparted by the *nem1*Δ and *pah1*Δ mutations (1, 36, 37, 42, 43).

Whereas the main function of Nem1-Spo7 is to control the phosphorylation state of Pah1, the phosphatase complex itself is subject to regulation by phosphorylation. Both subunits of Nem1-Spo7 are phosphorylated by protein kinase A (44) and protein kinase C (23). These kinases have opposing effects on the protein phosphatase function such as TAG synthesis; protein kinase A inhibits the Nem1-Spo7 phosphatase activity (44), whereas protein kinase C stimulates the enzyme activity (23). Moreover, the Nem1-Spo7 phosphatase is stimulated by the Pah1 substrate PA (45) but inhibited by the ER-associated protein Ice2 (46).

Previous work has shown that the C-terminal half of Nem1 is required for interaction with Spo7 (1). Given that Spo7’s interaction with Nem1 is mediated through few residues within CR1, CR2, and CR3 raised the possibility that a smaller segment of the C-terminal half might be sufficient for complex formation with Spo7. Using structural bioinformatics and site-specific mutagenesis, we identified eight conserved hydrophobic residues of Nem1 in the C-terminal region (CTR) necessary to form a phosphatase complex with Spo7 that is required for its physiological functions. This work advances the understanding of the molecular determinants of Nem1–Spo7 complex formation and its role in the activation of Pah1 PA phosphatase to regulate lipid synthesis.

## Results

### Nem1 requires its CTR hydrophobic residues to form a complex with Spo7

**Figure 2:**
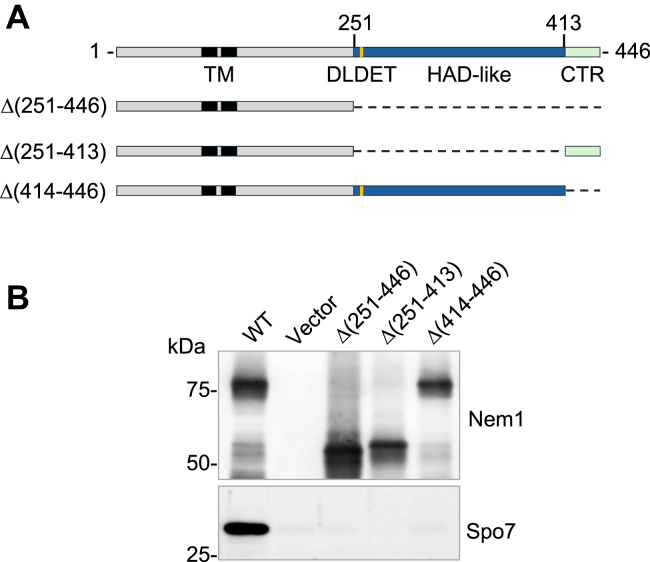
**Nem1 C-terminal truncation analysis of complex formation with Spo7.***A*, the diagram illustrates schematics of Nem1 with its transmembrane (TM) region, HAD-like domain with the DLDET catalytic motif, and CTR, along with the mutant truncation variants. *B*, cell extracts were prepared from *nem1*Δ *spo7*Δ *pah1*Δ (GHY85) cells co-expressing YCplac111-*GAL1/10*-*NEM1*-PtA (WT or mutant derivatives) and pRS314-*GAL1/10-SPO7*. The cell extracts were used for the isolation of protein A-tagged Nem1 proteins by IgG-Sepharose affinity chromatography, resolved by SDS-PAGE (12.5% polyacrylamide gel), and transferred to a polyvinylidene difluoride membrane. Sample loading was normalized to the elution volume of purified Nem1-PtA. The polyvinylidene difluoride membrane was split into the upper Nem1 and lower Spo7 portions and probed with rabbit anti-protein A and rabbit anti-Spo7 antibodies, respectively. The formation of the Nem1*–*Spo7 complex was scored by the presence of Spo7 in the affinity-purified Nem1 preparation (11). The positions of molecular mass standards are indicated. Data shown are representative of four replicate experiments. CTR, C-terminal region; HAD, haloacid dehalogenase.

The C-terminal half of Nem1 (residues 251–446) spans 196 amino acids with moderate sequence identity with 100% conservation in certain 8 to 10 residue blocks (1) (Fig. 2*A*). It contains the haloacid dehalogenase (HAD)–like domain (residues 251–413) with the catalytic active site motif DLDET and a CTR (residues 414–446) that flanks the catalytic domain (Fig. 2*A*). It remains unclear whether the entire C-terminal half is required for complex formation with Spo7 or if a smaller segment can mediate the interaction. To address this, we used site-directed mutagenesis to construct the mutant alleles of *NEM1* that produce variants that lack the C-terminal half (Δ(251–446)), the HAD-like domain (Δ(251–413)), and CTR (Δ(414–446)) of the protein (Fig. 2*A*). Each of the protein A-tagged Nem1 truncation variants was co-overexpressed with Spo7 in *nem1*Δ *spo7*Δ *pah1*Δ cells. The *pah1*Δ mutation in the expression strain was necessary to circumvent lethality caused by the overexpression of the Nem1–Spo7 complex in Pah1-expressing cells (10, 39). To determine which Nem1 variant abolishes complex formation with Spo7, we purified the catalytic subunits by affinity chromatography and examined the presence of the regulatory subunit (1, 11, 37, 42). Compared with WT Nem1, which was purified in association with Spo7, its C-terminal truncation variants did not associate with the regulatory subunit (Fig. 2*B*). This result indicated that the CTR of Nem1 is required for interaction with Spo7, but the data also indicated that the HAD-like domain of Nem1 is involved with the interaction of the subunits (Fig. 2*B*).

**Figure 3:**
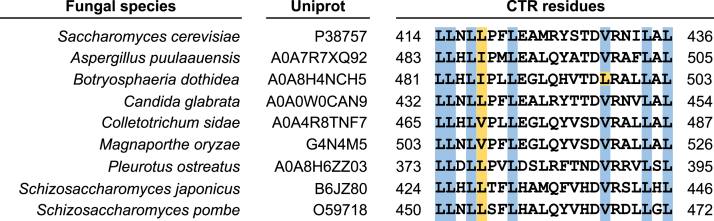
**CTR sequence alignment of *S. cerevisiae* Nem1 with its fungal orthologs.** The CTR residues of *S. cerevisiae* Nem1 and its fungal orthologs, which are shown with their UniProt accession numbers, are aligned by the Clustal Omega program. The conserved residues with identical and similar amino acids are highlighted in *blue* and *yellow*, respectively. Residue numbers are indicated at the start and end of each sequence. CTR, C-terminal region.

**Figure 4:**
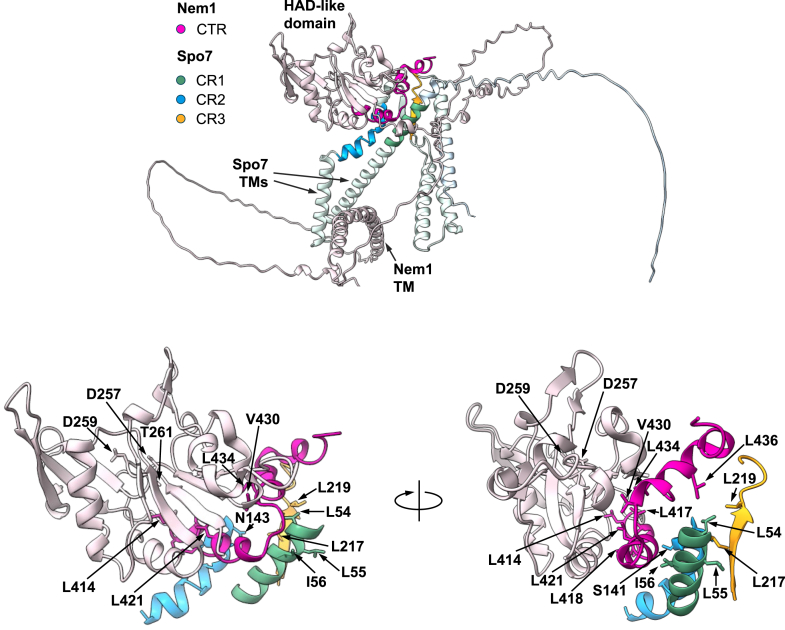
**AlphaFold-predicted structure of the Nem1*–*Spo7 complex.** The structure of the *S. cerevisiae* Nem1*–*Spo7 complex predicted by Alphafold is visualized by the UCSF ChimeraX program. The cartoon representations of the Nem1 and Spo7 structures are colored in *lavender* and *light blue*, respectively. The *callouts* zoom in on the CTR and HAD-like domain of Nem1 and CR1, CR2, and CR3 of Spo7. The residues experimentally shown to affect Nem1–Spo7 complex formation and the Nem1 catalytic site residues (D257, D259, and T261) are indicated. CTR, C-terminal region; CR, conserved region; TM, transmembrane region; HAD, haloacid dehalogenase.

To direct mutagenesis studies in the absence of experimentally determined structures for Nem1-Spo7, we utilized multiple sequence alignment of Nem1 and its fungal orthologs and AlphaFold structure prediction of the complex (Figs. 3 and 4, respectively). The multiple sequence alignment revealed that the CTR is rich in hydrophobic amino acids, specifically seven leucine and one valine that are conserved across other Nem1 orthologs (Fig. 3). AlphaFold predicts CTR (residues 414–446) forms a helical structure that is in close proximity to Spo7 conserved regions (*i.e.*, CR1, CR2, and CR3) whose hydrophobic residues are required for the complex formation with Nem1 (36, 37) (Fig. 4). The HAD-like domain (residues 251–413) is predicted to form a globular structure that is distinct from the CTR (Fig. 4). The domain/regions of Nem1-Spo7 are apart from their transmembrane regions that are responsible for membrane association (1) (Fig. 4). Nem1 and Spo7 form an obligate complex (1), the interaction of which is required for its protein phosphatase activity and stability (10, 36, 37, 46, 47). As hydrophobic interactions at the protein–protein interface promote the stabilization of protein complexes (48, 49, 50, 51, 52), we hypothesized that CTR hydrophobic residues of Nem1 are involved in forming the complex with Spo7.

**Figure 5:**
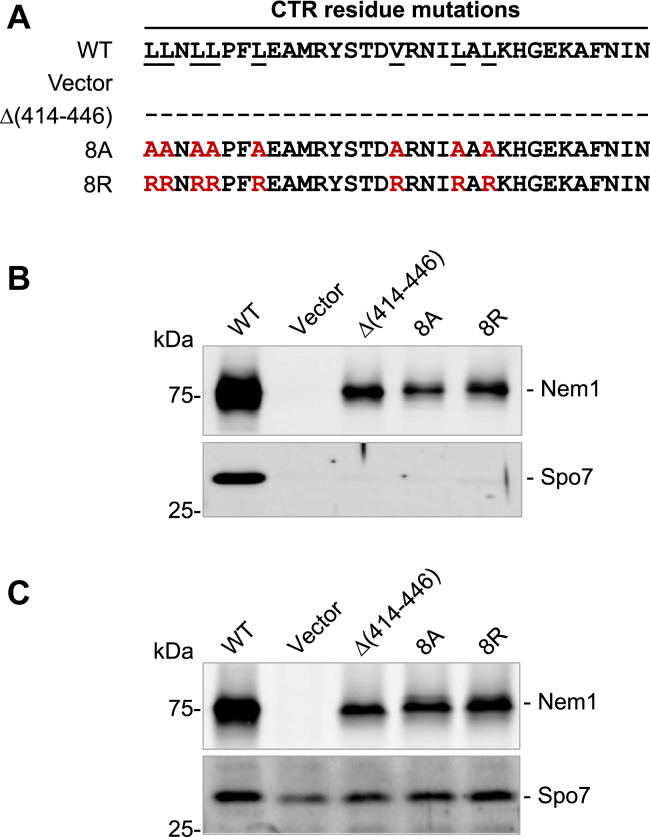
**The CTR hydrophobic residue variants of Nem1 do not form a complex with Spo7.***A*, the amino acid residues of Nem1 and its variants in the CTR are shown, and the eight alanine (8A) or arginine (8R) residues substituted for WT residues (*underlined*) are depicted in *red*. Cell extracts were prepared from *nem1*Δ *spo7*Δ *pah1*Δ (GHY85) cells co-expressing YCplac111-*GAL1/10*-*NEM1*-PtA (WT or mutant derivatives) and pRS314-*GAL1/10-SPO7*. The cell extracts were used for the isolation of protein A-tagged Nem1 proteins by IgG-Sepharose affinity chromatography or centrifuged at 100,000*g* for 1 h to isolate membranes. *B–C,* the affinity-purified Nem1 preparations (*B*) and the membranes (*C*) were resolved by SDS-PAGE (12.5% polyacrylamide gel) and transferred to a polyvinylidene difluoride membrane. Sample loading was normalized to the elution volume of purified Nem1-PtA (*B*) or to total membrane protein (30 μg) (*C*). The polyvinylidene difluoride membrane was split into the upper Nem1 and lower Spo7 portions and probed with rabbit anti-protein A and rabbit anti-Spo7 antibodies, respectively. The formation of the Nem1–Spo7 complex (*B*) was scored by the presence of Spo7 in the affinity-purified Nem1 preparation (11). The positions of Nem1, Spo7, and molecular mass standards are indicated. Data shown are representative of four replicate experiments. CTR, C-terminal region.

**Figure 6:**
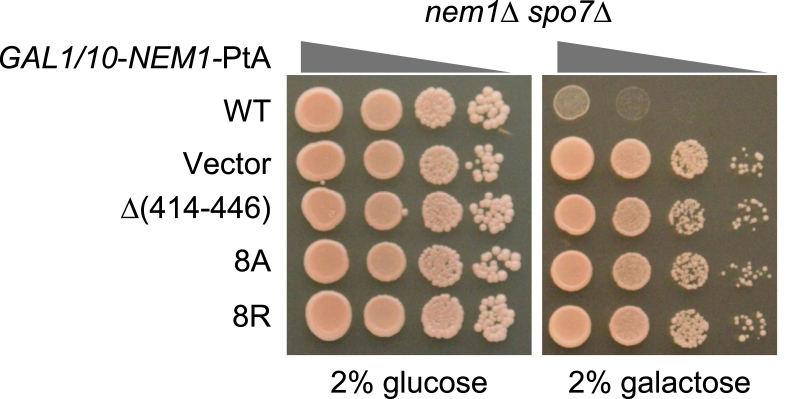
**Overexpression of the Nem1 CTR variant and Spo7 does not cause the lethality of the Pah1-harboring cells.** The *nem1*Δ *spo7*Δ (SS1010) cells were transformed with YCplac111-GAL1/10*-NEM1*-PtA (WT or mutant derivatives) and pRS314-GAL1/10*-SPO7*. The transformants were grown overnight to saturation in SC-Leu-Trp medium with 2% glucose as carbon source. The cells were washed with water and resuspended in the growth medium containing 2% glucose or 2% galactose. The cell suspension was adjusted to the absorbance of 0.67 at 600 nm, serially diluted (10-fold), and spotted onto SC-Leu-Trp agar plates containing 2% glucose or galactose. Cell growth was scored after incubation for 3 days at 30 ^o^C. The data are representative of three replicate experiments. CTR, C-terminal region.

To address this hypothesis, we constructed the mutant alleles of *NEM1* that produce the CTR alanine substituent 8A and the arginine substituent 8R for the hydrophobic amino acid residues (Fig. 5*A*). The alanine substitutions were used to conserve hydrophobicity in the CTR while minimizing potential large side chain interactions, whereas the arginine substitutions were used to introduce hydrophilicity to provide a significant contrast in property. Thus, we could assess how changes in the hydrophobicity or charge within CTR would affect Nem1–Spo7 complex formation and function. As discussed above, the protein A-tagged Nem1 mutants and Spo7 were co-overexpressed and purified from cells with the *nem1*Δ *spo7*Δ *pah1*Δ mutations, and the complex formation was scored by the presence of Spo7 in the affinity-purified preparations of the protein A-tagged Nem1 variants. The Nem1 CTR 8A and 8R mutant proteins did not associate with Spo7 (Fig. 5*B*). This result indicates that the CTR hydrophobic residues are required to form a complex with Spo7. The expression of Nem1 (WT or mutant forms) and Spo7 was confirmed from the membrane fraction by immunoblot analysis with anti-protein A and anti-Spo7 antibodies, respectively (Fig. 5*C*). Compared with WT Nem1, its variants (*i.e.*, Δ(414–446), 8A, and 8R) were detected at lower levels. This observation is consistent with previous studies indicating that Nem1 is stabilized through its complex formation with Spo7 (36, 37, 46, 47). The lack of Nem1–Spo7 complex formation caused by the CTR mutations obviated the lethality caused by the co-overexpression of Nem1 and Spo7 in Pah1-containing cells (10, 39) (Fig. 6).

### Nem1 phosphatase activity on Pah1 requires its CTR hydrophobic residues

**Figure 7:**
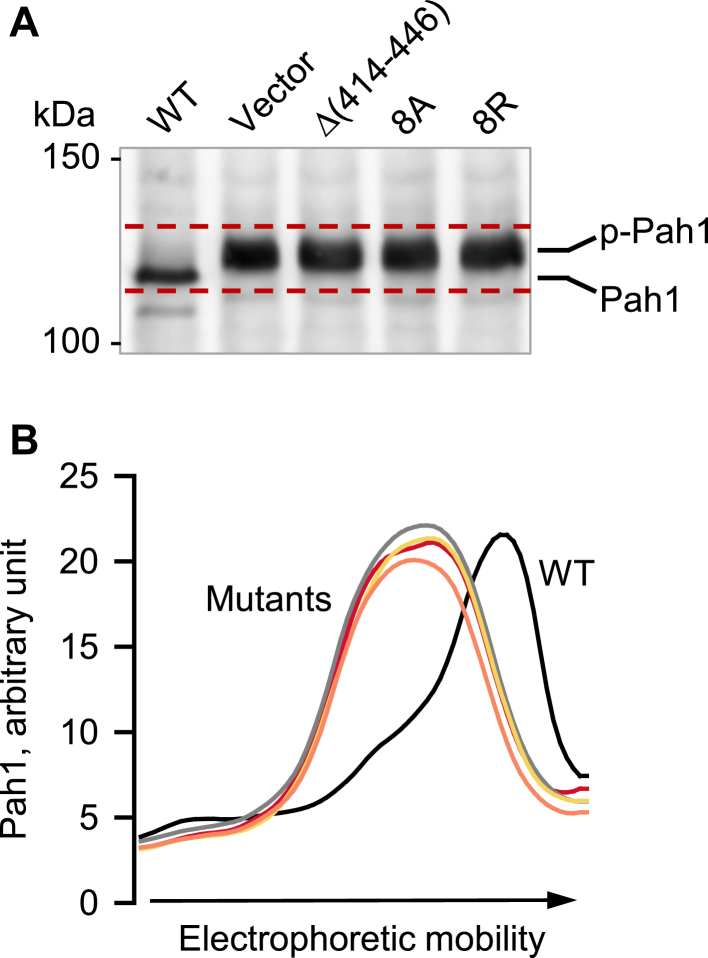
**Effect of the Nem1 CTR mutations on the Nem1-Spo7**–**mediated dephosphorylation of Pah1.** Under assay conditions for Nem1-Spo7 activity (11), purified phosphorylated Pah1 (2.5 ng) was incubated for 20 min at 30 ^o^C with the membranes (20 μg) prepared from *nem1*Δ *spo7*Δ *pah1*Δ (GHY85) cells co-expressing plasmids YCplac111-*GAL1/10-NEM1*-PtA (WT or mutant derivatives) and pRS314-*GAL1/10-SPO7*. *A,* following the incubation, the reaction mixtures were resolved by SDS-PAGE (6% polyacrylamide gel), transferred to a polyvinylidene difluoride membrane, and probed with anti-Pah1 antibody. The positions of Pah1 in the phosphorylated (*p-Pah1*) and dephosphorylated (*Pah1*) states are indicated with molecular mass standards. The *dashed red* line indicates the electrophoretic migration range of Pah1 after incubation with the membranes containing Nem1 or its variant and Spo7. *B,* the data are representative of triplicate experiments. The densitogram of Pah1 along its migration was produced by the line graph function of ImageQuant software. *Black*, WT; *red*, vector; *gray*, Δ(414–446); *yellow*, 8A; *orange*, 8R. CTR, C-terminal region.

Nem1, which forms a complex with Spo7, functions as a catalytic subunit to dephosphorylate Pah1 (10, 37, 42). Accordingly, we examined whether the C-terminal variants of Nem1 are active on the Pah1 substrate. Pah1 purified as a phosphorylated form was incubated with the membranes from the Pah1-deficient cells expressing Spo7 and the Nem1 variant (*i.e.*, Δ(414–446), 8A, or 8R) and then examined for its electrophoretic mobility in SDS-PAGE (11, 36, 37, 42). As described previously (11, 36, 37, 42), Pah1 showed a faster electrophoretic mobility with a concomitant decrease in protein abundance when it was incubated with WT Nem1 and Spo7 (Fig. 7). The decreased level of the faster-migrating Pah1 is attributed to its dephosphorylation, which renders the enzyme susceptible to proteolytic degradation (53, 54). In contrast to WT Nem1, its C-terminal variants Δ(414–446), 8A, and 8R did not increase the electrophoretic mobility of Pah1 (Fig. 7), indicating that the CTR hydrophobic residues of Nem1 are required for its phosphatase activity on Pah1.

### Nem1 requires its CTR hydrophobic residues for the translocation of Pah1 to the membrane

**Figure 8:**
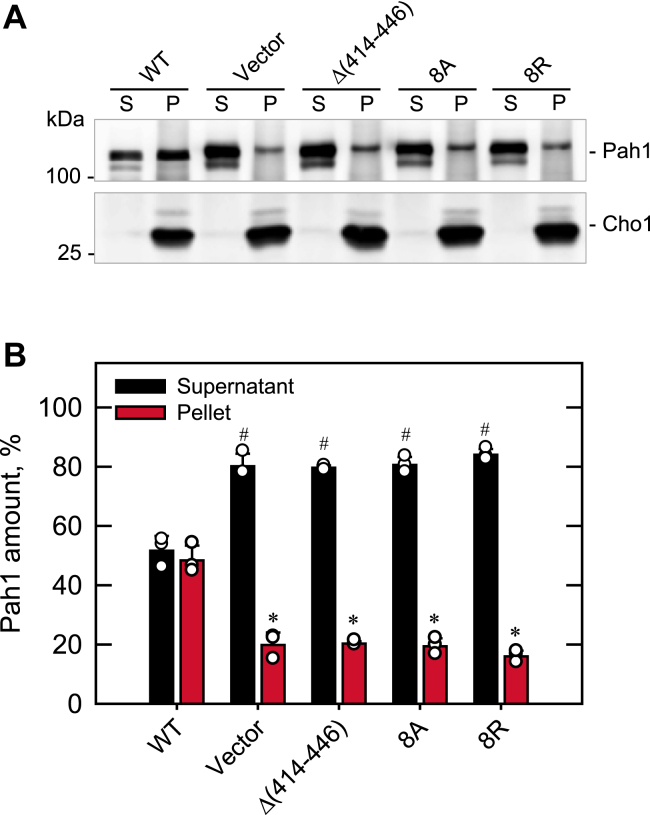
**Effect of the Nem1 CTR mutations on the Nem1-Spo7**–**mediated translocation of Pah1 to membranes.** Purified phosphorylated Pah1 (20 ng) was incubated for 20 min at 30 ^o^C in a total volume of 20 μl with the membranes (30 μg) prepared from *nem1*Δ *spo7*Δ *pah1*Δ (GHY85) cells co-expressing the plasmids YCplac111-*GAL1/10*-*NEM1*-PtA (WT and mutant derivatives) and pRS314-*GAL1/10-SPO7*. Following the incubation, the reaction mixture was centrifuged at 100,000*g* for 1 h at 4 °C. *A*, the membrane pellet (*P*) was resuspended to the volume of the supernatant (*S*), and equal proportions of the pellet and supernatant fractions were resolved by SDS-PAGE (10% polyacrylamide gel) and transferred to a polyvinylidene difluoride membrane, followed by probing with anti-Pah1 and anti-Cho1 (ER membrane marker) antibodies. The positions of Pah1, Cho1, and molecular mass standards are indicated. The data shown in *A* are representative of three independent experiments. *B*, the relative amount of Pah1 in the cellular fractions was determined by ImageQuant analysis of the three replicate experiments ± SD (*error bars*). The individual data points are also shown. ^#^*p* < 0.05 *versus* WT supernatant. ∗*p* < 0.05 *versus* WT membrane. CTR, C-terminal region.

The translocation of phosphorylated Pah1 to the nuclear/ER membrane is dependent on the Nem1–Spo7 complex (10, 11, 37, 55, 56). To examine the importance of the Nem1 CTR hydrophobic residues in this process, the purified phosphorylated Pah1 was incubated with Nem1-Spo7-containing membranes, followed by fractionation into the supernatant and pellet fractions (Fig. 8). For this assay, Nem1 and Spo7 were expressed in the *nem1*Δ *spo7*Δ cells harboring the *pah1*Δ mutation to obviate interference from endogenous Pah1. The analysis of Cho1 (ER marker) with anti-Cho1 antibody indicated a highly enriched membrane fraction. About 50% of Pah1 associated with the membrane containing WT Nem1 and Spo7 and showed a faster electrophoretic mobility as an indication of its dephosphorylation. In contrast, ≤ 20% of Pah1 associated with the membrane containing the Nem1 C-terminal variant (Δ(414–446), 8A, or 8R), which was similar to that associated with the membrane lacking Nem1 (vector control) and showed no change in electrophoretic mobility (Fig. 8). The accumulation of Pah1 in the membrane containing WT Nem1 and Spo7 was the result of the dephosphorylation-mediated translocation to the membrane. In addition, it is proposed that the faster-migrating Pah1 in the supernatant is the one that was dephosphorylated and subsequently dissociated from the membrane (Fig. 8*A*).

### Physiological functions of Nem1 require its CTR hydrophobic residues

The physiological functions of the Nem1–Spo7 complex are based on its role to recruit and dephosphorylate Pah1 and thereby activate PA phosphatase activity at the nuclear/ER membrane (12). Accordingly, cells lacking the Nem1–Spo7 complex display the Pah1-deficient phenotypes, which include a decrease in the levels of TAG and lipid droplets that is accompanied by an increase in the phospholipid content and the PA-mediated derepression of the genes encoding phospholipid biosynthetic enzymes (10, 12, 32, 33, 36, 37, 42, 57). We examined the properties of the *nem1*Δ cells expressing the WT Nem1 or its variants to determine the importance of the C-terminal hydrophobic residues.

#### Lipid composition

**Figure 9:**
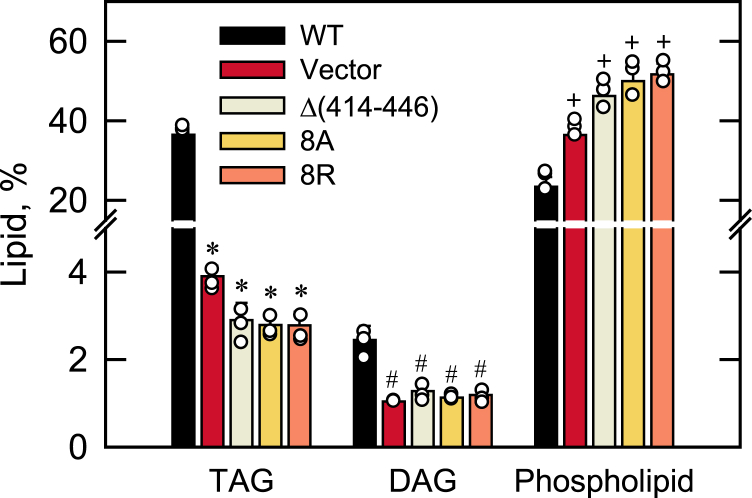
**Effect of the Nem1 CTR mutations on the Nem1-Spo7**–**mediated regulation of lipid composition.** The *nem1*Δ (WMY161) cells expressing YCplac111-*NEM1* (WT or mutant derivatives) were grown to the stationary phase in SC-Leu medium containing [2-^14^C]acetate (1 μCi/ml) at 30 ^o^C. Lipids extracted from the radiolabeled cells were separated by TLC, subjected to phosphorimaging, and quantified by ImageQuant analysis. The levels of TAG, DAG, and phospholipids were normalized to total chloroform-soluble lipids. The data are means ± SD (*error bars*) from three independent experiments. The individual data points are also shown. ∗*p* < 0.05 *versus* WT TAG. ^#^*p* < 0.05 *versus* WT DAG. +*p* < 0.05 *versus* WT phospholipids. CTR, C-terminal region; DAG, diacylglycerol; TAG, triacylglycerol.

To determine lipid composition, the *nem1*Δ cells expressing the Nem1 or its C-terminal variants Δ(414–446), 8A, and 8R were grown with [2-^14^C]acetate to the stationary phase when TAG levels are highest (2, 12, 32). The TLC analysis of the radiolabeled lipids indicated that the TAG, DAG, and phospholipid levels of the *nem1*Δ cells expressing WT Nem1 accounted for 36.5, 2.4, and 23.4%, respectively, of total lipids (Fig. 9). The relative amounts of these lipids were significantly affected by the CTR mutations. Compared with *nem1*Δ cells expressing Nem1, those expressing its C-terminal variants contained 12- and 2-fold lower levels of TAG and DAG, respectively, and a 2-fold higher level of phospholipids (Fig. 9). These results indicate that the CTR mutations abolish the *in vivo* function of Nem1-Spo7 in regulating lipid composition.

#### Lipid droplet formation

**Figure 10:**
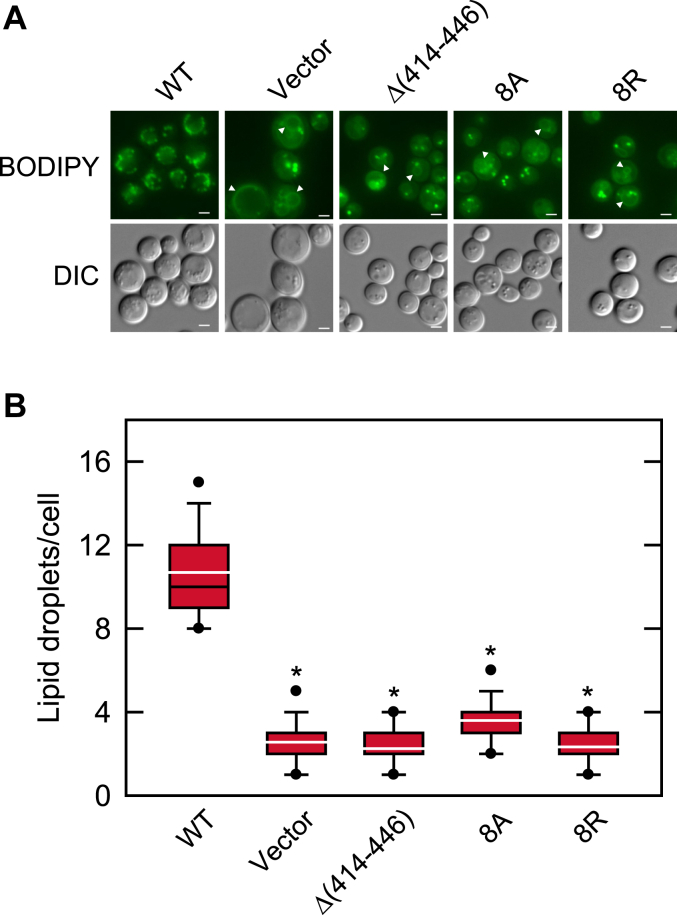
**Effect of the Nem1 CTR mutations on the Nem1-Spo7**–**mediated regulation of lipid droplet formation.** The *nem1*Δ (WMY161) cells expressing plasmid YCplac111-*NEM1* (WT or mutant derivatives) were grown at 30 ^o^C in SC-Leu medium to the stationary phase and then stained with BODIPY 493/503. *A,* the stained lipid droplets were visualized by fluorescence microscopy and counted from ≥ 200 cells (≥4 fields of view). The *white arrowheads* shown in the mutant images indicate membrane lipid staining (4, 33). *B,* the lipid droplet data are presented by the box plot. The *black* and *white lines* are the median and mean values, respectively, and the *black circles* are the outlier data points of the 5th and 95th percentile. The images shown in panel *A* are representative of multiple fields of view. *White bar*, 1 μm. ∗*p* < 0.05 *versus* WT. DIC, differential interference contrast; CTR, C-terminal region.

The cytoplasmic lipid droplet is the cellular organelle that originates from the nuclear/ER and stores TAG derived from the DAG produced by Pah1 PA phosphatase (4, 33, 58). Because the *nem1*Δ cells expressing the Nem1 C-terminal variants do not increase the TAG and DAG levels, they were examined for cytoplasmic lipid droplets by staining with the fluorescent lipophilic dye BODIPY 493/503 (Fig. 10*A*). The number of lipid droplets in the *nem1*Δ cells expressing the Nem1 variants was lower by 2.5- to 3.3-fold when compared with those expressing the WT protein (Fig. 10*B*). As described previously (4, 33), the cells defective in the Nem1-Spo7/Pah1 phosphatase cascade with reduced lipid droplet numbers exhibited BODIPY 493/503 staining of intracellular membranes (Fig. 10*A* , *white arrows*). The basis for the membrane staining is attributed to the neutral lipid accumulation in the ER membrane caused by a defect in Nem1-Spo7/Pah1 cascade function (33, 36, 42, 59).

#### CHO1 expression

**Figure 11:**
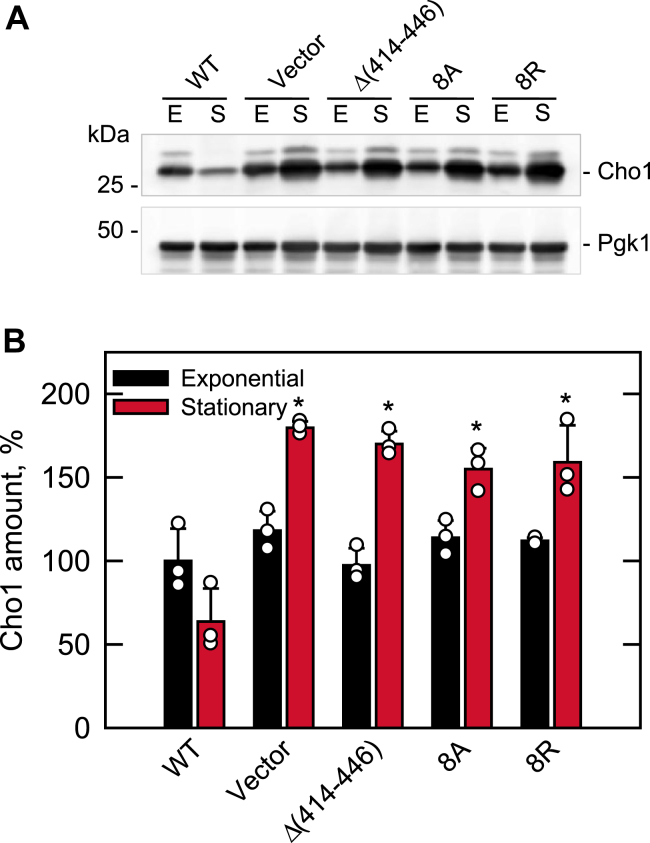
**Effect of the Nem1 CTR mutations on the Nem1-Spo7**–**mediated regulation of Cho1 expression.** The *nem1*Δ (WMY161) cells expressing plasmid YCplac111-*NEM1* (WT or mutant derivatives) were grown at 30 ^o^C in SC-Leu medium to the exponential (*E*) and stationary (*S*) phases. *A*, cell extracts were resolved by SDS-PAGE and transferred to a polyvinylidene difluoride membrane, which was probed for Cho1 and Pgk1 (loading control) with rabbit anti-Cho1 and mouse anti-Pgk1 antibodies, respectively (*panel A*). Sample loading was normalized to total protein (10 μg). The positions of Cho1 and Pgk1 are indicated with molecular mass standards. The Western blots are representative of three independent experiments. *B*, the relative amount of Cho1 was determined by ImageQuant analysis of the replicate experiments ± SD (*error bars*). The individual data points are also shown. ∗*p* < 0.05 *versus* WT stationary phase. CTR, C-terminal region.

The increased phospholipid synthesis in the *nem1*Δ cells expressing the C-terminal variants of Nem1 is caused, at least partially, by the PA-mediated derepression of phospholipid synthesis genes *via* the Henry (Opi1/Ino2-Ino4) regulatory circuit (10, 14, 15, 16, 17, 38, 60). To address this regulation, the level of the *CHO1*-encoded phosphatidylserine synthase, one of highly regulated phospholipid biosynthetic enzymes (14, 15, 57), was examined in the exponential and stationary phases of growth (Fig. 11). In WT cells, *CHO1* expression is higher in the exponential phase when phospholipid synthesis predominates over TAG synthesis, whereas its expression is lower in the stationary phase when TAG synthesis predominates over phospholipid synthesis (57, 61, 62, 63, 64, 65). The Cho1 level in *nem1*Δ cells expressing WT Nem1 was 1.6-fold lower in the stationary phase when compared with the exponential phase (Fig. 11). As expected, the lack of Nem1-Spo7/Pah1 phosphatase cascade function (vector control) removed the growth phase–mediated *CHO1* repression (Fig. 11). In the stationary phase, the Cho1 level in the *nem1*Δ cells was 2.8-fold higher when compared with those expressing WT Nem1 (Fig. 11). The Nem1 C-terminal variants expressed in the *nem1*Δ cells failed to correct the aberrant derepression phenotype in the stationary phase as the Cho1 level was higher (2.4- to 2.7-fold) when compared with that of *nem1*Δ cells expressing WT Nem1 (Fig. 11).

## Discussion

The Nem1–Spo7 complex, which functions as protein phosphatase to activate Pah1 PA phosphatase at the nuclear/ER membrane, plays an important role in the regulation of the PA-DAG balance for yeast lipid synthesis (1, 10, 11, 36, 37, 38, 39, 40, 41). For its enzyme activity and protein stability, the Nem1 catalytic subunit requires the regulatory subunit Spo7, which recruits the protein substrate (1, 10, 11, 36, 37, 38, 39, 40, 41, 46, 47). In the formation of the obligate complex, Nem1 is known to interact with Spo7 through its C-terminal half (residues 251–446) (1), which constitutes the HAD-like catalytic domain and a CTR (Fig. 2*A*). In this work, we showed that the CTR of Nem1, which flanks the catalytic domain, is necessary to form a complex with Spo7. The CTR is highly conserved in fungal organisms, specifically in the hydrophobic amino acids, Leu and Val (Fig. 3). Its deletion disrupted interaction with Spo7. We identified eight hydrophobic residues within the CTR that are critical for interaction with Spo7. The alanine and arginine substitutions for the hydrophobic residues did not allow the interaction of Nem1 with Spo7 (Fig. 5*B*). The prevention of the complex formation by the alanine substitutions, which mimic the hydrophobicity of the CTR residues, indicates that the side chain properties of the hydrophobic residues, leucine and valine, are crucial for the protein–protein interactions.

The Nem1-interacting subunit Spo7 contains three conserved homology regions (CR1, CR2, and CR3) (66). Mutations in any one of the regions are sufficient to disrupt the complex formation with Nem1 and its catalytic function (36, 37). The defect of Spo7 in the complex formation is caused by mutational effects on the hydrophobicity of LLI (residues 54–56) within CR1, the uncharged hydrophilicity of STN (residues 141–143) within CR2, or hydrophobicity of LL (residues 217 and 219) within CR3 (36, 37). In AlphaFold (13, 67, 68) predicted structure of the Nem1–Spo7 complex, the CTR of the catalytic subunit forms a helix that is positioned in close proximity to the conserved homology regions (CR1, CR2, and CR3) of Spo7 (Fig. 4). The predicted structure of Nem1-Spo7 and mutational effects of the conserved CTR hydrophobic residues indicate that hydrophobic interaction is a major factor in the formation of the protein phosphatase complex.

The AlphaFold structure of Nem1-Spo7 indicates that the Nem1 CTR is distinct and appears sandwiched in between the Spo7 conserved regions and the HAD-like domain (Fig. 4). The proximity of the CTR residues with residues of the Spo7 conserved regions provides an explanation for the mutational effects on Nem1–Spo7 complex formation. For example, L436 of CTR is in proximity to L54 of CR1 and L219 of CR3 (Fig. 4, *callouts*). Interestingly, AlphaFold predicts that at least four of the eight CTR hydrophobic residues (L414, L417, L421, and V430) are in proximity to the Nem1 HAD-like domain (Fig. 4, *callouts*). In deletion analysis, loss of the HAD-like domain disrupted interaction with Spo7. Because CTR residues may interact with the HAD-like domain, we posit that removal of the domain altered the conformation of CTR preventing its interaction with Spo7. The predicted structure also shows that some residues within one conserved region are in proximity to residues in another conserved region (*e.g.*, L54 of CR1 and L219 of CR3; N143 of CR2 and L217 of CR3) providing an explanation for why a mutation in any one of the Spo7 conserved regions disrupts complex formation with Nem1 (36, 37) (Fig. 4, *callouts*).

During the course of this work, Ren *et al.* (69) confirmed that components of the Nem1-Spo7/Pah1 phosphatase cascade in the fungal pathogen *Botryosphaeria dothidea* are functionally similar to the cascade components in *S. cerevisiae*. Additionally, they showed that the truncation of the CTR of the *B. dothidea* Nem1 prevented its interaction with Spo7 (69). This work, however, lacked information on the residues that constitute the CTR or the hydrophobic residues required for the interaction with Spo7 that are reported here. Moreover, studies to assess the physiological relevance of the CTR-mediated interaction of Nem1 with Spo7 were not addressed for the *B. dothidea* proteins that are addressed here.

The Nem1-Spo7/Pah1 phosphatase cascade conserved in mammals is known as CTDNEP1 (formerly Dullard)-NEP1R1 (formerly TMEM188)/lipin 1 (66, 70, 71). As in *S. cerevisiae*, the CTDNEP1–NEP1-R1 complex catalyzes the dephosphorylation of lipin 1 to regulate its PA phosphatase function (66, 71). The disruption of the mammalian phosphatase cascade also results in a host of deleterious lipinopathies, revealing the important role of lipin 1 activity (72, 73, 74, 75, 76, 77, 78, 79). In regard to the CTDNEP1 protein phosphatase, studies have shown that it plays a crucial role in the regulation of nuclear/ER membrane biogenesis (71, 80, 81); maintenance of nuclear membrane stability during interphase (82); nuclear pore complex insertion (83); regulation of nuclear positioning during cell migration (84, 85); and maintenance of genomic stability by regulating mitotic chromosome segregation (80, 86). CTDNEP1 has been identified as a tumor suppressor in highly aggressive Myc-driven medulloblastoma, and its deficiency is associated with poor patient prognosis (86), presenting a targetable therapeutic opportunity for drug discovery. As in yeast, the importance of the CTDNEP1-NEP1-R1/lipin 1 phosphatase cascade is most likely stemming from its role in regulating the PA-DAG balance.

CTDNEP1 depends on NEP1-R1 for its stability and function to limit the nuclear/ER membrane expansion (87). Recent structural- and AlphaFold-based analyses have shown that like yeast Nem1 and Spo7, CTDNEP1 and NEP1-R1 interact through a hydrophobic interface (87). M220, A223, and V233 in CTDNEP1 and F30 in CR1 of NEP1-R1 have been shown to be key residues for the protein–protein interaction (81, 87). However, there is no indication that CTDNEP1 interacts with CR2 and CR3 of NEP1-R1. In a complex formed from CTDNEP1 and a soluble version of NEP1-R1, the two conserved regions are disordered (81). Although the residues involved in the interaction of CTDNEP1 and NEP1-R1 are different from those of the yeast Nem1 (this work) and Spo7 (36), hydrophobic interaction for the complex formation is conserved in the CTR of the catalytic subunit and CR1 of the regulatory subunit (81).

While yeast Nem1 and mammalian CTDNEP1 show the conservation of the CTR, they show poor sequence similarity in the N-terminal region. Structural bioinformatics predict that Nem1 contains an N-terminal transmembrane region (Fig. 4), and deletion of this region prevents its insertion into the membrane (1). A peripheral membrane association of Nem1 without its transmembrane region may be afforded by its complex formation with Spo7 that contains a transmembrane region that is retained in the membrane (1). Experimental evidence (1), in conjunction with the AlphaFold structure prediction (Fig. 4), indicates that the CTR-mediated interaction of Nem1 with Spo7 occurs in the cytosol. In contrast, AlphaFold predicts that CTDNEP1 does not possess the N-terminal transmembrane region but instead contains an amphipathic helix; deletion of the helix disrupts its association with a model membrane (81). Additionally, for the recruitment and activation of Pah1 by the Nem1–Spo7 complex at the nuclear/ER membrane, the basic tail of Spo7 interacts with the acidic tail of Pah1 (40, 42). Mammalian NEP1-R1 and lipin 1 do not contain the analogous C-terminal sequences, and underlying mechanism for the activation of lipin 1 by CTDNEP1-NEP1-R1 is unclear.

## Experimental procedures

### Reagents

All chemicals utilized were of reagent grade or higher. The growth media were procured from Difco Laboratories. DNA and plasmid extraction kits were obtained from Qiagen. Carrier DNA and In-Fusion HD cloning kit were sourced from Clontech. New England Biolabs provided the enzyme reagents necessary for DNA manipulation along with the Q5 site-directed mutagenesis kit. Reagents for Western blotting, Bradford protein assay, and molecular mass protein standards were procured from Bio-Rad. Polyvinylidene difluoride membrane, enhanced chemifluorescence substrate for Western blotting, and IgG-Sepharose beads were sourced from GE Healthcare. Millipore-Sigma supplied Silica gel 60 TLC plates, ampicillin, bovine serum albumin, 2-mercaptoethanol, PCR primers for mutagenesis, nucleotides, Triton X-100, and protease inhibitors. Thermo Fisher Scientific provided BODIPY 493/503. Radiochemicals were procured from Revvity, and scintillation-counting supplies were obtained from National Diagnostics.

### Antibodies

Rabbit polyclonal antibodies were generated against the amino acid sequence EDDLRRQAHEQK (residues 58–69) of Spo7 (37), TSIDKEFKKLSVSKAGA (residues 778–794) of Pah1 (56), and MVESDEDFAPQEFPH (residues 1–15) of Cho1 (88). The IgG fraction of each antiserum was purified (89) and used for immunoblot analyses. Millipore-Sigma supplied rabbit anti-protein A antibody (product number P3775, lot 025K4777) and goat anti-mouse IgG antibody conjugated with alkaline phosphatase (product number A3562, lot number SLBG1482 V). Abcam was the source of mouse anti-Pgk1 antibody (product number ab113687, lot number 2101050637). Thermo Fisher Scientific provided goat anti-rabbit IgG antibody conjugated with alkaline phosphatase (product number 31340, lot number NJ178812).

### Plasmids and DNA manipulations

The plasmids used in this study are listed in Table 1. Plasmid DNA was isolated and manipulated using standard protocols (93, 95, 96). PCR primers were designed with the online NEBaseChanger program. Q5 site-directed mutagenesis was utilized unless otherwise indicated. Transformation of *E.coli* DH5α (93) and *S. cerevisiae* strains (97) with plasmid DNA was performed using conventional methods. YCplac111-*NEM1*, which directs the low-copy expression of *NEM1* from its native promoter, was constructed by deleting the protein A-tag from YCplac111-*NEM1*-PtA. The Δ(251–446), Δ(251–413), and Δ(414–446), derivatives of YCplac111-based plasmids, were generated by deletion of the codons corresponding to 251 through 446, 251 through 413, and 414 through 446, respectively, of Nem1. The *NEM1* DNA fragments containing the 8A and 8R mutations were synthesized by Genewiz and inserted into the YCplac111-based vectors using In-Fusion HD cloning kits. All mutations were confirmed by DNA sequencing. Plasmid pGH452 bearing *PAH1*-PtA under the control of *GAL1/10* promoter was used for the purification of phosphorylated Pah1 (92).

**Table 1:** Plasmids and strains used in this study

Plasmid or strain	Relevant characteristics	Source or reference
Plasmid		
YCplac111	Single-copy number *E. coli*/yeast shuttle vector with *LEU2*	(90)
*Derivatives*		
YCplac111-*NEM1*-PtA	*NEM1*-PtA with native promoter in YCplac111	S. Siniossoglou
YCplac111-*NEM1*	*NEM1* with native promoter in YCplac111	This study
YCplac111-*NEM1*-Δ(414–446)	YCplac111-*NEM1* lacking residues 414–446	This study
YCplac111-*NEM1*-8A	YCplac111-*NEM1* with the L414 A/L415 A/L417 A/L418 A/L421 A/V430 A/L434 A/L436 A mutations	This study
YCplac111-*NEM1*-8R	YCplac111-*NEM1* with the L414 R/L415 R/L417 R/L418 R/L421 R/V430 R/L434 R/L436 R mutations	This study
YCplac111-*GAL1/10*-*NEM1*-PtA	*NEM1*-PtA with *GAL1/10* promoter in YCplac111	(1)
YCplac111-*GAL1/10*-*NEM1*-Δ(251–446)-PtA	YCplac111-*GAL1/10*-*NEM1*-PtA lacking residues 251–446	This study
YCplac111-*GAL1/10*-*NEM1*-Δ(251–413)-PtA	YCplac111-*GAL1/10*-*NEM1*-PtA lacking residues 251–413	This study
YCplac111-*GAL1/10*-*NEM1*-Δ(414–446)-PtA	YCplac111-*GAL1/10*-*NEM1*-PtA lacking residues 414–446	This study
YCplac111-*GAL1/10*-*NEM1*-8A-PtA	YCplac111-*GAL1/10*-*NEM1*-PtA with the L414 A/L415 A/L417 A/L418 A/L421 A/V430 A/L434 A/L436 A mutations	This study
YCplac111-*GAL1/10*-*NEM1*-8R-PtA	YCplac111-*GAL1/10*-*NEM1*-PtA with the L414 R/L415 R/L417 R/L418 R/L421 R/V430 R/L434 R/L436 R mutations	This study
pRS314	Single-copy number *E. coli*/yeast shuttle vector with *TRP1*	(91)
Derivative		
pRS314-*GAL1/10-SPO7*	*SPO7* with *GAL1/10* promoter in pRS314	(44)
pGH452	*PAH1-*PtA with *GAL1* promoter in pYES2	(92)
Strain		
***E.coli***		
DH5α	F^-^ ϕ80d*lacZ*ΔΜ15Δ (*lacZYA*-*argF*)U169 *deoR rec*A1 *end*A1 *hsd*R17 (*r*_k_^-^*m*_*k*_^+^) *pho*A *sup*E44 λ^−^*thi-*1 *gyr*A96 *rel*A1	(93)
*S*. *cerevisiae*		
RS453	*MAT***a** *ade2-1 his3-11,15 leu2-3112 trp1-1 ura3-52*	(94)
Derivatives		
WMY161	*nem1*Δ*::URA3*	(44)
SS1010	*nem1::HIS3 spo7::HIS3*	(1)
GHY85	*nem1::HIS3 spo7::HIS3 pah1*Δ*::natMX4*	(37)
SS1132	*nem1*Δ*::HIS3 pah1*Δ*::TRP1*	(56)

### Strains and growth conditions

The bacterial and yeast strains used in this work are listed in Table 1. Plasmid amplification and maintenance were performed with *Escherichia coli* strain DH5α. The DH5α cells were grown at 37 °C in Luria-Bertani broth medium (1% tryptone, 0.5% yeast extract, and 1% NaCl, pH 7.0) containing 100 μg/ml ampicillin to select transformants carrying plasmids. The *nem1*Δ*::URA3* strain (WMY161) was used for low-copy expression of WT and Nem1 mutant proteins. The *nem1*Δ *spo7*Δ *pah1*Δ strain (GHY85) was used for the galactose-induced overexpression of the protein A-tagged Nem1 and its variants and Spo7. The *pah1*Δ mutation in GHY85 prevents lethality caused by the overexpression of the Nem1–Spo7 protein phosphatase complex (10). The *pah1*Δ *nem1*Δ strain (SS1132) was used for the pGH452-mediated overexpression and purification of Pah1. The *nem1*Δ mutation in strain SS1132, which lack the Nem1–Spo7 complex, ensures the hyperphosphorylation of Pah1 (10, 92).

Conventional methods were employed for culturing *S. cerevisiae* (95). Cells were grown at 30 °C in YPD medium (1% yeast extract, 2% peptone, and 2% glucose) or synthetic complete (SC) medium containing 2% glucose. The plasmid-containing cells were grown in synthetic dropout media without leucine (SC-Leu) or without leucine and tryptophan (SC-Leu-Trp). Cell growth in liquid media was estimated spectrophotometrically by measuring absorbance at 600 nm (*A*_*600*_). For lipid analysis, *nem1*Δ cells expressing WT or mutant forms of Nem1 were grown in synthetic dropout media to the exponential phase (*A*_*600*_ ∼ 0.5) and then grown for an additional 24 h to the stationary phase. For galactose induction of Nem1-PtA (WT and mutant forms) and Spo7, cells were inoculated at a final cell density of *A*_*600*_ ∼ 0.1 and grown overnight in SC-Leu-Trp (2% glucose) to saturation. The saturated culture was harvested by centrifugation at 1500*g* for 10 min, washed with water, and resuspended in SC-Leu-Trp (2% galactose and 1% raffinose) media at a final cell density of A_600_ ∼ 0.4 and incubated for 14 h (*A*_*600*_ ∼ 0.4–0.5) with shaking at 250 rpm.

### Lipid labeling and analysis

The steady-state labeling of cellular lipids with [2-^14^C]acetate (98), lipid extraction (99), and one-dimensional TLC analysis using hexane/diethyl ether/glacial acetic acid (40:10:1, v/v) (100) were performed as described by Fakas *et al.* (101). The resolved lipids on silica gel 60 plates were visualized by phosphorimaging with the Storm 860 Molecular Imager (GE Healthcare) and quantified by ImageQuant software using a standard curve of [2-^14^C]acetate. The identity of the radiolabeled lipids was confirmed by comigration of authentic standards visualized by iodine vapor staining.

### Lipid droplet analysis

*S. cerevisiae* cells were cultured at 30 °C in SC-Leu media to the stationary phase, incubated with the fluorescent dye BODIPY 493/503 (1 μg/ml) for 30 min, washed twice with phosphate buffered saline (pH 7.4), and resuspended in the same buffer for fluorescence microscopy (36, 37, 42). The fluorescent signal from the lipid droplets was examined under a Nikon Eclipse Ni-U microscope equipped with an EGFP/FITC/Cy2/AlexaFluor 488 filter. The images were captured by a DS-Qi2 camera and analyzed with the NIS-elements BR software. The number of lipid droplets per cell was determined by examination from ≥ 4 fields of view (≥200 cells).

### Preparation of cell extracts, membranes, and protein isolations

All steps were conducted at 4 ^o^C. Yeast cultures were harvested by centrifugation at 1500*g* for 10 min, cells were washed with water, and resuspended in lysis buffer (50 mM Tris-HCl [pH 7.5], 10% glycerol, 10 mM 2-mercaptoethanol, 1 mM EDTA, 0.5 mM phenylmethylsulfonyl fluoride, 1 mM benzamidine, 5 μg/ml aprotinin, 5 μg/ml leupeptin, and 5 μg/ml pepstatin). Cell lysis was achieved by mechanical disruption with glass beads (0.5-mm diameter) using a BioSpec Products Mini-Beadbeater-16 (five repeats of 1-min burst, followed by 2-min cooling) (102). Lysates were centrifuged at 1500*g* for 10 min to separate unbroken cells and cell debris (pellet) from the cell extract (supernatant). The total membrane fraction was collected from the cell extract by centrifugation at 100,000*g* for 1 h (102); the membranes were suspended in EDTA-free lysis buffer.

Protein A-tagged Nem1–Spo7 complex was isolated from cell extracts (3 mg protein) of *nem1*Δ *spo7*Δ *pah1*Δ (GHY85) cells expressing plasmids YCplac111-*GAL1/10*-*NEM1*-PtA (WT or mutant forms) and pRS314-*GAL 1/10-SPO7* using IgG-Sepharose affinity chromatography (1, 11). The amount of the complex proteins isolated was not sufficient for detection by Coomassie Blue protein staining on an SDS-polyacrylamide gel. Instead, isolated Nem1-PtA and Spo7 proteins were identified by Western blotting using anti-protein A and anti-Spo7 antibodies, respectively. Phosphorylated Pah1 was isolated from the *pah1*Δ *nem1*Δ mutant (SS1132) cells expressing plasmid pGH452 employing a combination of IgG-Sepharose chromatography, anion exchange chromatography, and size-exclusion chromatography (92). The purity of the Pah1 preparation matched that reported previously (92). All protein preparations were stored at -80 °C.

### SDS-PAGE and Western blotting

Standard procedures were followed for SDS-PAGE (103) and Western blotting (104, 105). Normalized gel loading was confirmed by protein concentration determination by the method of Bradford (106) using bovine serum albumin as a standard. Protein transfer from the polyacrylamide gel to the polyvinylidene difluoride membranes was monitored using Ponceau S staining. Blotting membranes were probed with rabbit anti-protein A (1 μg/ml), rabbit anti-Spo7 (2 μg/ml), rabbit anti-Pah1 (2 μg/ml), or rabbit anti-Cho1 (0.25 μg/ml) primary antibodies. The secondary alkaline phosphatase-conjugated goat anti-rabbit IgG antibody and goat anti-mouse IgG antibody were used at a dilution of 1:5000. An enhanced chemifluorescence substrate for alkaline phosphatase was utilized for the detection of the immune complexes. Fluorescence signals from immunoblots were acquired with a Storm 860 Molecular Imager (GE Healthcare). The signal intensities were analyzed by ImageQuant TL software (GE Healthcare).

### Nem1-Spo7 protein phosphatase assay

The protein phosphatase activity of Nem1-Spo7 was assessed by examining the electrophoretic mobility of Pah1 on 6% polyacrylamide gels as described previously (36, 37, 42). The reaction mixture contained 100 mM sodium acetate (pH 5.0), 10 mM MgCl_2_, 1 mM DTT, 20 μg membranes, and 2.5 ng Pah1 in a total volume of 20 μl. The phosphorylated and dephosphorylated forms of Pah1 were visualized by Western blotting with anti-Pah1 antibody.

### Predicted structure analysis

AlphaFold (13, 67, 68) predicted structures of the *S. cerevisiae* Nem1–Spo7 complex were visualized and imaged using UCSF ChimeraX software (107, 108, 109).

### Data analysis

Statistical analyses were performed with Microsoft Excel software. *p* values < 0.05 were taken as a significant difference.

## Data availability

All data are contained within the manuscript.

## Conflict of interest

The authors declare that they have no conflicts of interest with the contents of this article.
